# Preparation of hydrogen, fluorine and chlorine doped and co-doped titanium dioxide photocatalysts: a theoretical and experimental approach

**DOI:** 10.1038/s41598-021-81979-x

**Published:** 2021-03-11

**Authors:** Petros-Panagis Filippatos, Anastasia Soultati, Nikolaos Kelaidis, Christos Petaroudis, Anastasia-Antonia Alivisatou, Charalampos Drivas, Stella Kennou, Eleni Agapaki, Georgios Charalampidis, Abd. Rashid bin Mohd Yusoff, Nektarios N. Lathiotakis, Athanassios G. Coutsolelos, Dimitris Davazoglou, Maria Vasilopoulou, Alexander Chroneos

**Affiliations:** 1 Institute of Nanoscience and Nanotechnology (INN), National Center for Scientific Research Demokritos, Agia Paraskevi, 15310 Athens, Greece; 2 Faculty of Engineering, Environment and Computing, Coventry University, Priory Street, Coventry, CV1 5FB UK; 3 Theoretical and Physical Chemistry Institute, National Hellenic Research Foundation, Vass. Constantinou 48, 11635 Athens, Greece; 4 Department of Electrical and Electronics Engineering, Faculty of Engineering, University of West Attica, Campus 2, No. 250, Thivon str., 12244 Athens, Greece; 5 School of Mining and Metallurgical Engineering, National Technical University of Athens, 9 Iroon Polytechniou Str., Zografou Campus, 15780 Athens, Greece; 6 Department of Chemical Engineering, University of Patras, 26504 Patras, Greece; 7 Department of Chemistry, University of Crete, Laboratory of Bioinorganic Chemistry, Voutes Campus, P.O. Box 2208, 71003 Heraklion, Crete Greece; 8 Department of Physics, Vivian Tower, Singleton Park, Swansea University, Swansea, SA2 8PP UK; 9 Department of Materials, Imperial College, London, SW7 2AZ UK

**Keywords:** Materials for energy and catalysis, Electronic properties and materials

## Abstract

Titanium dioxide (TiO_2_) has a strong photocatalytic activity in the ultra-violet part of the spectrum combined with excellent chemical stability and abundance. However, its photocatalytic efficiency is prohibited by limited absorption within the visible range derived from its wide band gap value and the presence of charge trapping states located at the band edges, which act as electron–hole recombination centers. Herein, we modify the band gap and improve the optical properties of TiO_2_ via co-doping with hydrogen and halogen. The present density functional theory (DFT) calculations indicate that hydrogen is incorporated in interstitial sites while fluorine and chlorine can be inserted both as interstitial and oxygen substitutional defects. To investigate the synergy of dopants in TiO_2_ experimental characterization techniques such as Fourier transform infrared (FTIR), X-ray diffraction (XRD), X-ray and ultra-violet photoelectron spectroscopy (XPS/UPS), UV–Vis absorption and scanning electron microscopy (SEM) measurements, have been conducted. The observations suggest that the oxide’s band gap is reduced upon halogen doping, particularly for chlorine, making this material promising for energy harvesting devices. The studies on hydrogen production ability of these materials support the enhanced hydrogen production rates for chlorine doped (Cl:TiO_2_) and hydrogenated (H:TiO_2_) oxides compared to the pristine TiO_2_ reference.

## Introduction

Transition metal oxides such as titanium dioxide (TiO_2_) have been drawn large attention as photocatalysts due to their high chemical stability and strong ultra-violet (UV) absorption^1–10^. The anatase phase of TiO_2,_ in particular, exhibits higher photocatalytic activity than the rutile and brookite ones, which, however, is restricted due to its wide band gap (3.2 eV) and the surfac and bulk trap state mediated electron–hole recombination^7^. For a photocatalyst to reach high efficiency, the band gap has to be approximately 2.0 eV, whereas the band gap edges have to be in agreement with the redox potential of water^11^. To improve the photochemical activity of TiO_2_ it is crucial to increase its UV–Vis absorption, reduce surface and bulk defects and increase charge transport in order to further suppress their capture in trap states.

An effective way to improve both the photochemical activity and the electrical properties of TiO_2_ is doping, for example, with transition metal or non-metal elements^12–18^. Fluorine (F) ion has been frequently reported as an effective dopant for metal oxides such as TiO_2_ and tin oxide (SnO_2_)^19, 20^, the electronic structure of which can be manipulated through the creation of gap states and the reduction of their band gap^21–24^. Previous studies have demonstrated that F also reduces the hole diffusion from the bulk to the surface of TiO_2_ and recombination therein^25–28^. Furthermore, an enhanced photocatalytic activity in fluorine doped TiO_2_ (termed as F:TiO_2_) was observed from the experiments of Yu et al*.*^27^. However, as the energy levels of F-2p orbitals are in a lower position than the O-2p ones, the reduction in the band gap value of TiO_2_ upon fluorine doping is inferior compared to other promising dopants such as nitrogen (N)^29–32^. The extra electrons that are added within the oxide system upon F doping have strong interaction with the 3d orbitals of Ti without contribution to free energy states below the band gap. As a result, the absorption of F:TiO_2_ in the visible region is still limited. Besides F, other halogens such as iodine (I) have also been considered as dopants in TiO_2_^33–35^. On the other hand, limited research has been conducted for chlorine (Cl) doped TiO_2_ with an emphasis on the synthesis and catalytic activity. Specifically, Xu et al*.*^36^, have prepared a Cl doped TiO_2_ material (termed as Cl:TiO_2_) and demonstrated its high potential for solar cell applications. Furthermore, DFT calculations have shown that Cl doping can significantly reduce the band gap of the bulk anatase TiO_2_ with the introduction of gap states which play an essential role to the device efficiency^24^.

Hydrogen (H) has a small ionic radius molecule (0.53 Å) and, therefore, can diffuse easily inside a crystal, especially in inorganic compounds, occupying interstitial sites. As a TiO_2_ dopant, H does not increase the crystal size significantly, while it can slightly reduce the band gap as it creates shallow donor sites when occupies an interstitial position^37, 38^. Recently, heavily H doped TiO_2_ (referred to as black titania) triggered a great scientific interest due to its high solar absorption and superior photocatalytic properties^39^. Furthermore, hydrogen treatment is a simple, pure and beneficial way to improve the surface properties of TiO_2_, without inducing any impurity energy states therein. Previous studies suggested that the enhanced visible light absorption is connected with the formation of Ti–H or Ti–OH bonds in a disorganized surface layer on the hydrogenated TiO_2_^40–42^. However, even though the enhanced absorption of H doped TiO_2_ (termed as H:TiO_2_), its photocatalytic activity under solar light irradiation remains low^43, 44^. Both X(X = F,Cl):TiO_2_ and H:TiO_2_ show improved optical properties, compared to the undoped anatase TiO_2_, so it is interesting to investigate whether and how these two treatment techniques can act synergistically and if they can further improve the photocatalytic properties.

To explore and expand the photocatalytic properties of TiO_2_, in this work, we have examined the effect of F and Cl doping of TiO_2_ before and after hydrogen annealing both experimentally and with DFT calculations. We focus on the band gap engineering of TiO_2_ and its correlation with the crystal structure, morphology, nature of bonding and optical properties combining DFT with various characterization techniques such as XRD sudies, UV–Vis, FTIR, XPS and UPS spectroscopies as well as SEM topographies. We show that F and Cl intercalation within the oxide lattice cause n-type doping with the creation of shallow states near the conduction band minimum (CBM) as well as deep intergap states. Moreover, H doping achieved through hydrogen annealing decreases the band gap as it creates available energy states near the valence band maximum (VBM). The incorporation of H both in the bulk and the surface hence forms shallow acceptors with Cl doped TiO_2_ having a significantly improved absorption, which significantly improves the hydrogen production rate compared to the pristine oxide. After analyzing our theoretical and experimental results, we propose a mechanism according to which Cl and F atom most favorably substitutes an oxygen atom in a vacancy, thus eliminating the electron trap therein.

## Results and discussion

### Computational results and discussion

#### Bulk anatase TiO_2_

**Figure 1:**
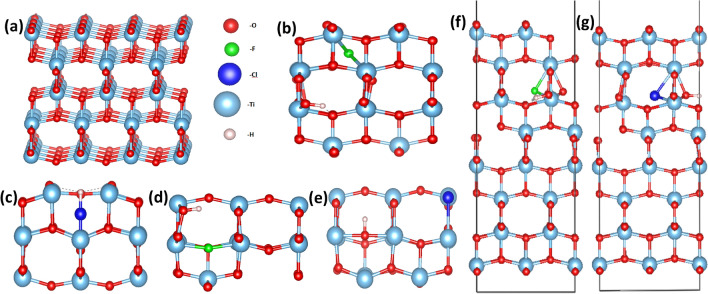
The structures of (**a**) plain TiO_2_ supercell, (**b**) F_i_:H_i_:TiO_2_, (**c**) Cl_i_:H_i_:TiO_2_, (**d**) F_o_:H_i_:TiO_2_ and (**e**) Cl_o_:Hi:TiO_2_. The surface structure of (**f**) F_i_:H_i_:TiO_2_ and (**g**) Cl_i_:H_i_:TiO_2._

**Figure 2:**
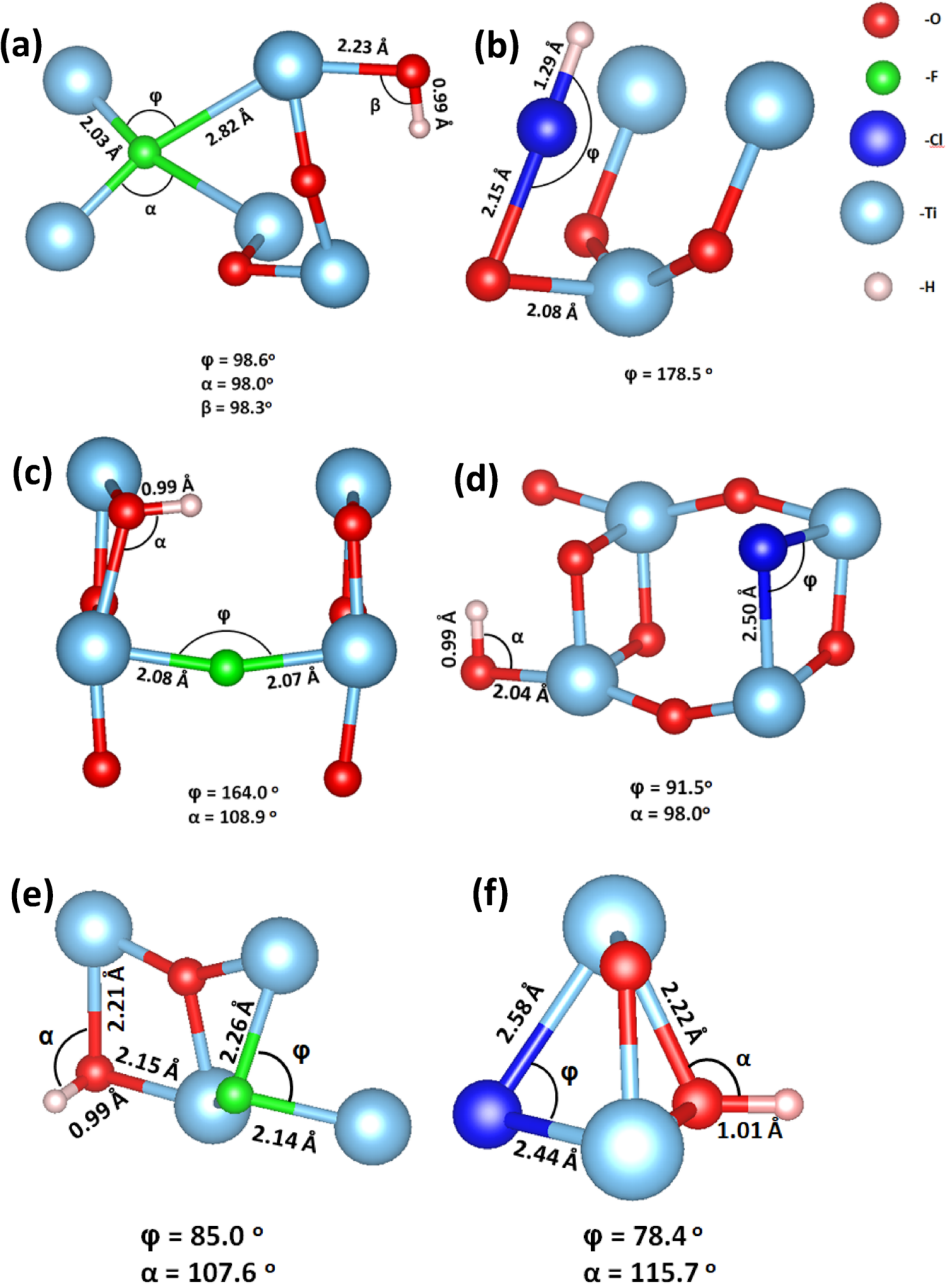
The structures, the angles and the distances of (**a**) F_i_:H_i_:TiO_2_, (**b**) Cl_i_:H_i_:TiO_2_, (**c**) F_o_:H_i_:TiO_2_ and (**d**) Cl_o_:Hi:TiO_2._ The surface structures with distances and angles for the (**e**) F_i_:H_i_:TiO_2_ and (**f**) Cl_i_:H_i_:TiO_2_.

**Figure 3:**
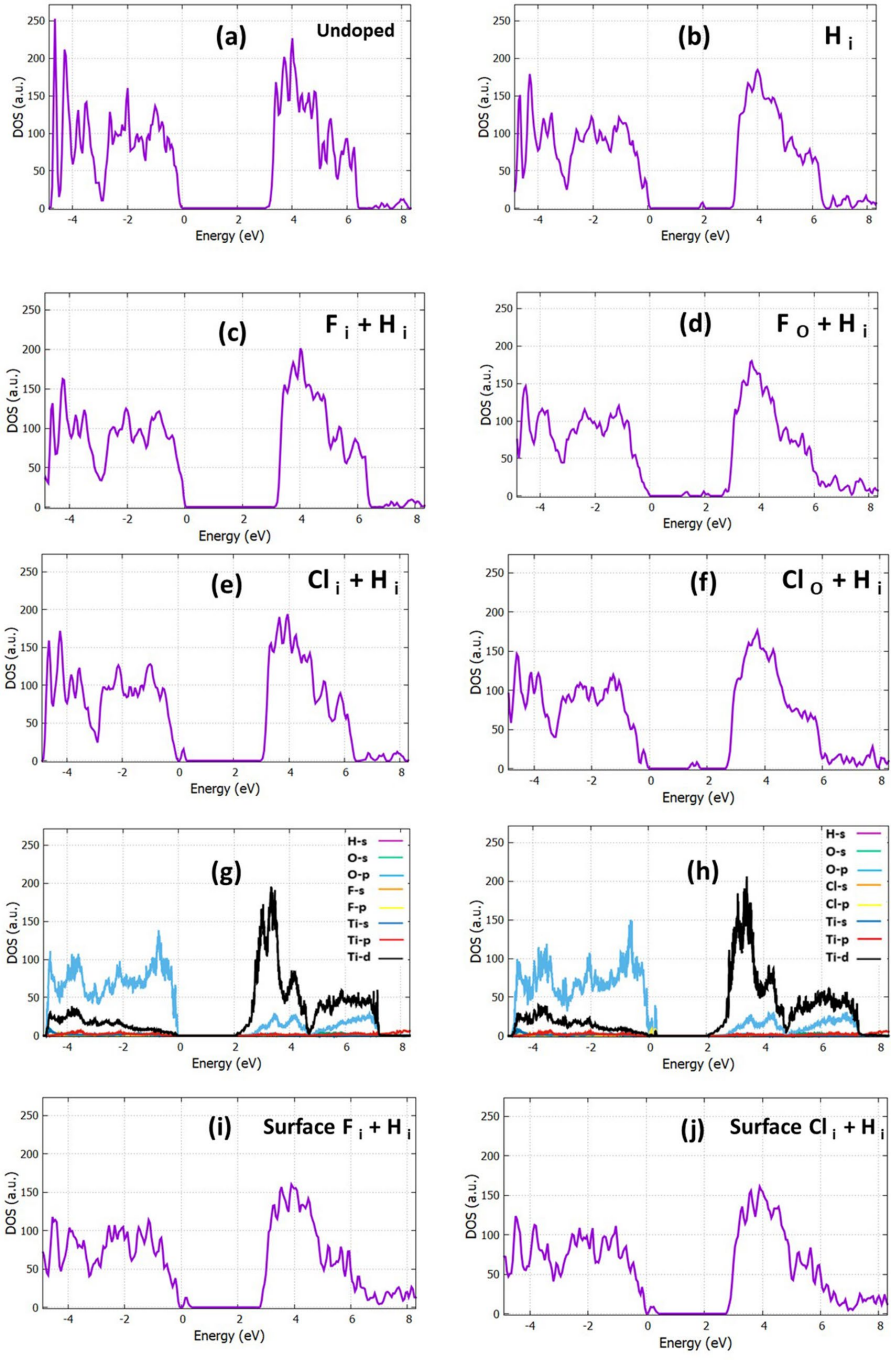
The DOS of (**a**) TiO_2_, (**b**) H_i_:TiO_2_, (**c**) F_i_:H_i_:TiO_2_, (**d**) F_o_:H_i_:TiO_2_, (**e**) Cl_i_:H_i_:TiO_2_ and (**f**) Cl_o_:H_i_:TiO_2._ The projected DOS of interstitial doped structures (**g**) F_i_:H_i_:TiO_2_ and (**h**) Cl_i_:H_i_:TiO_2_. The DOS of (**i**) F_i_:H_i_:TiO_2_ and (**j**) Cl_i_:H_i_:TiO_2_.

Between the three stable polymorphs of TiO_2_ (anatase, brookite, and rutile), the anatase is the most frequently studied for energy harvesting devices due to its superior photocatalytic properties^45^. The crystal structure for the anatase TiO_2_ is body-centered tetragonal with space group I4/amd. From diffraction techniques, the lattice parameters of the single crystal TiO_2_ are determined: a = b = 3.782 Å, and c = 9.502 Å^46^. Our structural theoretical calculations, which are performed in the geometry optimization scheme, predicted the lattice parameters at: a = b = 3.804 Å and c = 9.729 Å (Fig. 1a), which are in good agreement with the experimental and previous DFT results^47, 48^. In this work, we focus on point defect engineering processes with dopant concentrations of 2 dopant atoms per 108 TiO_2_ atoms for the bulk system and 2 dopant atoms per 96 TiO_2_ atoms for the surface system. From our DFT calculations derives that F and Cl dopants are stable either as interstitial defects or as oxygen substitutional defects in the bulk system. We have compared the energy of the relaxed structures of interstitial and substitutional doped TiO_2_ and we concluded that the substitutional doping of TiO_2_ has the lowest energy. As a result, it is energetically favorable for both F and Cl to substitute an oxygen atom rather than to occupy an interstitial position. In contrast, the small radius H, diffuses easier inside the crystal and it mostly occupies interstitial sites. As was indicated by Atei et al*.*^37^, in the case of stoichiometric crystal in thermodynamic equilibrium the most stable configuration for a H atom is the interstitial position. Studying systematically many defect formations in the bulk TiO_2_, we conclude that the minimum energy system for F_i_:H_i_:TiO_2_ and Cl_i_:H_i_:TiO_2_ are the ones shown in Fig. 1b,d. The simple substitution of oxygen atoms by F and Cl in the TiO_2_ lattice with H in interstitial position is shown in Fig. 1c,e, respectively. Distances and angles from the nearest oxygen atom for the relaxed structures, are shown in Fig. 2a–d. For Cl_i_:H_i_:TiO_2,_ the minimum energy system occurs when H and Cl form an HCl molecule inside the bulk. This can also be seen from the distance between the Cl and H atom (1.28 Å) in Fig. 2c, which is equal to the molecular bond length of HCl (1.27 Å). Figure 3a–f provides the DOS for each supercell. For our simulation, we use the DFT + U approximation, with the U parameter equal to 8.2 eV for the Ti-3d orbital. For the undoped case, we calculate a band gap equal to 3.14 eV (Fig. 3a), which agrees with other theoretical studies^49–51^, and experimental values (3.2 eV)^52^. The amount of doping, is calculated about 1.85% for the interstitial cases and 1.88% for the substitutional. This amount of doping is the smallest amount that can be used for a supercell without affecting converged lattice parameters that we predicted in our calculations.

Next, we performed a calculation with the H in the interstitial position. As shown in Fig. 3b, the calculated DOS of H_i_ is plotted, and it is seen that there is an occupied defect level at 1.0 eV below the CBM. This result also agrees with previous calculations^53–55^. As it is shown in Fig. 3c, the band gap of the F, H interstitially co-doped TiO_2_ reduces to 3 eV, which is equal to the experimental value predicted from Gao et al.^56^. In a previous work^24^, it was demonstrated that in the case of F:TiO_2_ mid gap states were created, and they behaved as shallow acceptors. However, for the co-doped F_i_:H_i_:TiO_2,_ we have no indication for the formation of mid gap states. To fully understand the contribution of H_i_ to F_i_:TiO_2_, the partial DOS was calculated (Fig. 3g). As we observe, H creates some available states inside the CB and thus behaves as an effective n-type dopant. In the case of the F_o_:H_i_:TiO_2_ we find that the band gap is reduced to 2.60 eV (Fig. 3d); moreover, some mid gap states are created at 1.46 eV as well as some available states near the conduction band at 2.20 eV. Focusing on the bulk Cl:H:TiO_2_, in the case of Cl_i_:H_i_:TiO_2_ (Fig. 3e) the DOS shows some energy levels near the VB at 0.20 eV. This is an indication that these dopants work as acceptors. Besides, it is seen that the band gap of the bulk TiO_2_ and Cl:TiO_2_ is significantly reduced to 2.85 eV. To analyze the energy levels, we present the PDOS (Fig. 3h), which indicates that these states are mainly created from the hybridization of O-2p, Cl-2p, orbitals. On the other hand, for the Cl_o_:H_i_:TiO_2_ the band gap is further reduced, and it reaches the value of 2.66 eV. In this case, the energy states are created near the minimum of the CB, indicating that the co-doped material works as an n-type conductor. This remarkable band gap narrowing in all these cases of co-doping may result in the enhancement of photon absorption under visible light irradiation.

#### Surface doping

For the development of better photocatalytic materials with high activity, selectivity, and visible light response, surface doping requires special attention. However, the investigation on the surface doping and the surface electronic structure are scarce compared to the studies concerning the bulk^57^. From XRD studies, it was deduced that the dominant facets for anatase TiO_2_ are the (101), (001), (010) and (100) planes^58–60^. Among them, (101) plane is the most frequently observed and the (001) is the more photocatalytically reactive^61^. As it was predicted by Lazzeri et al*.*^61^,the energy of the unreconstructed (001) surface of TiO_2_ is 0.9 J/m^2^, which is about 0.5 J/m^2^ higher than the energy of (101) surface.

Moreover, Liu et al*.*^62^ used a photoelectrochemical method and described the (001) plane of TiO_2_ as the plane with the highest photocatalytic activity. In a previous study, the (001) TiO_2_ surface is considered mainly for the interaction with water, oxygen, or even small molecules^63–65^. Gong et al*.*^63, 64^, studied the absorption of formic acid in TiO_2_ and found that it is highly favored in (001) plane, which also plays a vital role in the reactivity of TiO_2_ nanoparticles. Wang et al.^65^, studied the adsorption of Pt nanoparticles in TiO_2_ and found that when TiO_2_ exposes the (001) facets, then the material exhibits higher photocatalytic performance under visible light irradiation. It was determined that the reactivity of the (010) and (100) planes of TiO_2_ is better than that of the (101) surface but not as good as that of (001) surface^66^. All the above prove that the (001) facets of anatase titanium dioxide possess better photocatalytic activity than other facets and holds the potential for the improvement of the material for device application. Therefore, we select the (001) TiO_2_ surface for the DFT modeling. This section aims to demonstrate the changes in the electronic properties of the F:H:TiO_2_ (001) and the Cl:H:TiO_2_ (001) surface based on spin-polarized DFT + U calculations. We calculate the interatomic distances and angles as well as the DOS for the undoped and the co-doped structures. For our simulations, we use a vacuum of 14 Å in the (001) direction and apply periodic boundary conditions in the other directions. We keep fixed the bottom four layers to assimilate the bulk area while the top 4 layers were fully relaxed.

The relaxed structures of the F:H:TiO_2_ and Cl:H:TiO_2_ are shown in Fig. 1f,g, respectively. In Fig. 2e,f, the distances of the dopants to the nearest oxygen atom in the minimum energy systems are displayed. Focusing on the total DOS of each case (Fig. 3i,j), it is observed that the fluorine and hydrogen co-doped TiO_2_, as well as chlorine and hydrogen co-doped TiO_2_, have the same effect in the band gap of the surface (Fig. 3i,j respectively). Specifically, the band gap reaches a bigger value of 2.83 eV for (001) F:H:TiO_2_ and 2.77 eV for (001) Cl:H:TiO_2_ compared to the un-doped structure which exhibits a value of 2.36 eV. It is also observed that in the case of these co-doped materials, some energy levels near the valence band are created, proving that these co-dopants behave as acceptors. Therefore, after analyzing the DFT results, we get fundamental insights into the doping mechanism, and it was revealed that the insertion of F, Cl and H is seen to have a significant effect on the electrical properties of our system, both in bulk material and on its surface. We expect that from the interstitial and substitutional doping with F, Cl, and H atoms and the occupation of oxygen sites as well as the oxygen vacancies will result in the reduction of oxygen dangling bonds. This will have a positive effect on the device performance because the trap sites that are located inside the band gap will be reduced.

### Experimental study

#### Fourier transform infrared (FTIR) spectroscopy

**Figure 4:**
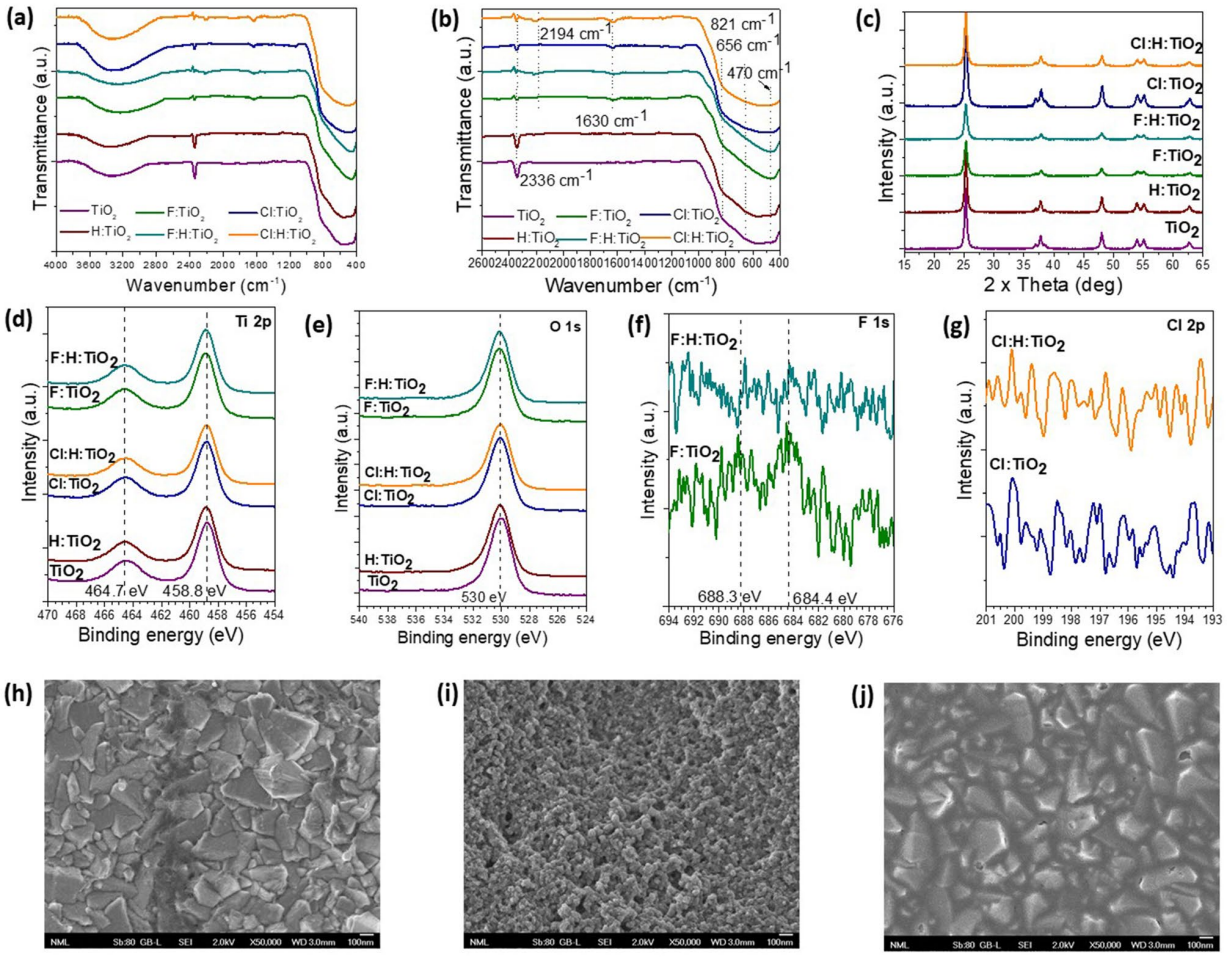
The FTIR transmittance spectra of pristine and F, Cl-doped TiO_2_ samples before and after hydrogen annealing: (**a**) The whole wavenumber region, (**b**) the short wavenumber region. (**c**) The XRD spectra of the same samples. XP spectra of the (**d**) Ti 2p and (**e**) O 1 s regions of all samples. (**f**) XP spectra of the F 1 s region of the F-doped and F,H-co-doped TiO_2_ samples. (**g**) XP spectra of the Cl 2p region of the Cl-doped and Cl,H–co-doped TiO_2_ samples. SEM topographic top view images of (**h**) pristine TiO_2_, (**i**) F-doped and (**j**) Cl-doped TiO_2_ layers.

Doped and co-doped samples were prepared as explained in detail in “Methods”. In brief, F and Cl doping was performed through the addition of ammonium fluoride (NH_4_F) and ammonium chloride (NH_4_Cl) water solutions in the TiO_2_ precursor. The dopant concentration was estimated about 0.7% w/w relative to TiO_2_. H doping was performed through annealing in hydrogen environment; the dopant concentration could not be estimated in this case. Six samples were prepared termed as: TiO_2_, H:TiO_2_, F:TiO_2_, Cl:TiO_2_, F:H:TiO_2_, Cl:H:TiO_2_. The co-doped samples were first doped with halogens and the resultant films were annealed in hydrogen environment. FTIR spectra of the prepared samples are shown in Fig. 4a for the 4000 cm^−1^ to 400 cm^−1^ wavenumber region; a zoom-in the 2600 cm^−1^ to 400 cm^−1^ wavenumber region is presented in Fig. 4b. In all cases, a broad band consisting of peaks at 470 and 656 and 821 cm^−1^ are due to TiO_2_ and specifically due to Ti–O and Ti–O–Ti bridging stretching vibrations. The reduction in the intensity of the peak at 656 cm^−1^ in the halogen doped samples is due to the substitution of Ti–O with Ti–X vibrations. An additional peak at 1630 cm^−1^ is observer in the halogen doped samples which is ascribed to N–H bending (which is due to the ammonium salts used to achieve halogen doping). The broad peaks at 3200–3600 cm^−1^ are due to the stretching vibration of the hydroxyl group (–OH), which suggests that all these surfaces are rich to –OH groups. The bands of the hydroxyl groups are broader in the halogen doped-TiO_2_ samples, which was previously considered beneficial for the oxide photocatalytic activity^67^. Moreover, this is an indication that the hydrogen atom can be more easily inserted within the oxide surface upon halogen co-doping. However, due to the contribution of the water, we cannot estimate the content of H dopants. The peak at 2400 cm^−1^ is created from stretching of O=C=O due to the absorption of carbon dioxide (CO_2_) from the atmosphere. The reduction of this peak upon halogen doping is consistent with the substitutional mechanism proposed here; as halogen atoms occupy oxygen vacancies the adsorption sites for CO_2_ are significantly reduced.

#### Structural characterization

The crystal phase of our six samples were analyzed by XRD. As it is shown in Fig. 4c, all our samples have the typical anatase XRD pattern with diffraction peaks at 25.2°, 37.8°, 48°, 53.9°, 55°,62.6°, 75.2° which corresponds to the (101), (004), (200), (105), (211), (213) and (215) facets, respectively. It is seen that the hydrogen annealing in the case of F:TiO_2_ gives rise to a (511) facet, and the XRD pattern of the Cl:H:TiO_2_ is similar to the XRD of the pure TiO_2_. We anticipated that this is because the diffusion of hydrogen is the case of Cl:TiO_2_ is limited within a few nanometers from the oxide surface. The difficulty of the diffusion of hydrogen inside Cl:TiO_2_ can be attributed to the big radius of chlorine (1.75 Å), which reduces the free space inside the bulk. Also, as it was calculated from the previous DFT simulation, the H-atom in the Cl:TiO_2_ system is of lowest energy when it is attracted by the Cl atoms. It is expected that this attraction might limit the diffusion of hydrogen inside the bulk. On the other hand, when fluorine occupies oxygen sites no significant structural changes are expected because the ionic radius of fluorine is almost similar to that of oxygen. When hydrogen is inserted, it is expected to diffuse easily inside the bulk structure, and as it is seen in the XRD pattern of F:H:TiO_2 ,_this is why new planes, such as (511), are created. Also, the crystallite sizes of the most dominant peaks were calculated using the Debye–Scherrer formula, and the results are presented in Table 1. Comparing the crystal sizes before and after the annealing in hydrogen containing environment, it is seen that hydrogen affected the crystallite size leading to an increase in most of the cases. The same result can also be conducted from the aforementioned DFT calculations as it is seen that in all the examined cases, when hydrogen is inserted, the average crystal size changes. This is beneficial for the photocatalytic activity of the material since larger crystallite size is equivalent to fewer grain boundaries where trap states for the photogenerated electron–hole pairs are usually located. By suppressing the number of grain boundaries, we can also suppress the undesired recombinations. It is important to note that from the XRD pattern, it is seen that the most significant increase from all the crystal sizes occurs in the 37.8°, which corresponds to the (001) plane. This proves that hydrogen atoms prefer mostly the (001) surface over the other facets.

**Table 1:** The crystal size of each structure as calculated with the Scherrer–Debye equation.

2 × Theta	TiO_2_ crystal size (nm)	H:TiO_2_ crystal size (nm)	Cl:TiO_2_ crystal size (nm)	F:TiO_2_ crystal size (nm)	Cl:H:TiO_2_ crystal size (nm)	F:H:TiO_2_ crystal size (nm)
25.2°	17.01	16.95	15.68	12.67	16.36	13.34
37.8°	9.32	17.42	6.71	8.30	8.74	11.54
48.0°	21.64	21.86	18.23	15.10	18.62	16.65
53.9°	20.83	21.29	18.69	16.32	18.74	18.05
55.0°	21.26	22.12	19.99	16.85	21.02	17.48
62.6°	20.46	22.18	19.38	17.18	20.54	17.23
75.2°	30.13	38.70	21.89	25.58	22.52	21.10

#### Compositional studies

We also investigated changes in composition upon doping of TiO_2_ samples by using X-ray photoelectron spectroscopy (XPS) measurements. Figure 4d shows the Ti 2p core levels of the XP spectra for all the samples. Ti 2p_3/2_ is located at a binding energy (BE) of 458.8 ± 0.1 eV with a spin orbit splitting (S.O.S.) of 5.7 eV, corresponding to TiO_2_^68^. No significant changes are observed upon the different treatments. Figure 4e shows the O 1 s region for all the samples. A wide peak is observed centred at 530.1 ± 0.1 eV, corresponding to O–Ti bonds and –OH groups adsorbed on the surface. No significant changes were observed at the position or the width of the photopeaks. Figure 4f presents the XPS spectra of the F 1 s region for the F doped samples. For the F:TiO_2_ sample, two peaks can be seen at 688.3 and 684.4 ± 0.1 eV attributed to F–Ti–O bonds and F^−^ adsorbed on the surface, respectively^69^. Upon the H_2_ treatment, no traces of F were detected. This was also the case for the Cl 2p region for the Cl doped samples as shown in Fig. 4g. No traces of Cl were detected for both samples. This is an indication that halogen dopants are inserted into the oxide’s lattice and occupy substitutional positions while their incorporation onto the surface to occupy dangling bonds is less possible. This is in agreement with our theoretical results explain above.

#### Surface morphology

We also investigated possible changes in the morphology in the doped samples. As seen in Fig. 4h–g where the scanning electron microscopy images of the pristine and halogen doped samples are presented, the F doped sample exhibits a pronounced different morphology compared to pristine and Cl doped samples. It consists of much smaller particles whereas the particle size in the Cl doped sample is even larger than the pristine one. Larger particles might be beneficial for the material’s photocatalytic activity as it increases the surface area and therefore the number of available sites for the catalytic hydrogen production.

#### UV–Vis absorption and energy gap values

**Figure 5:**
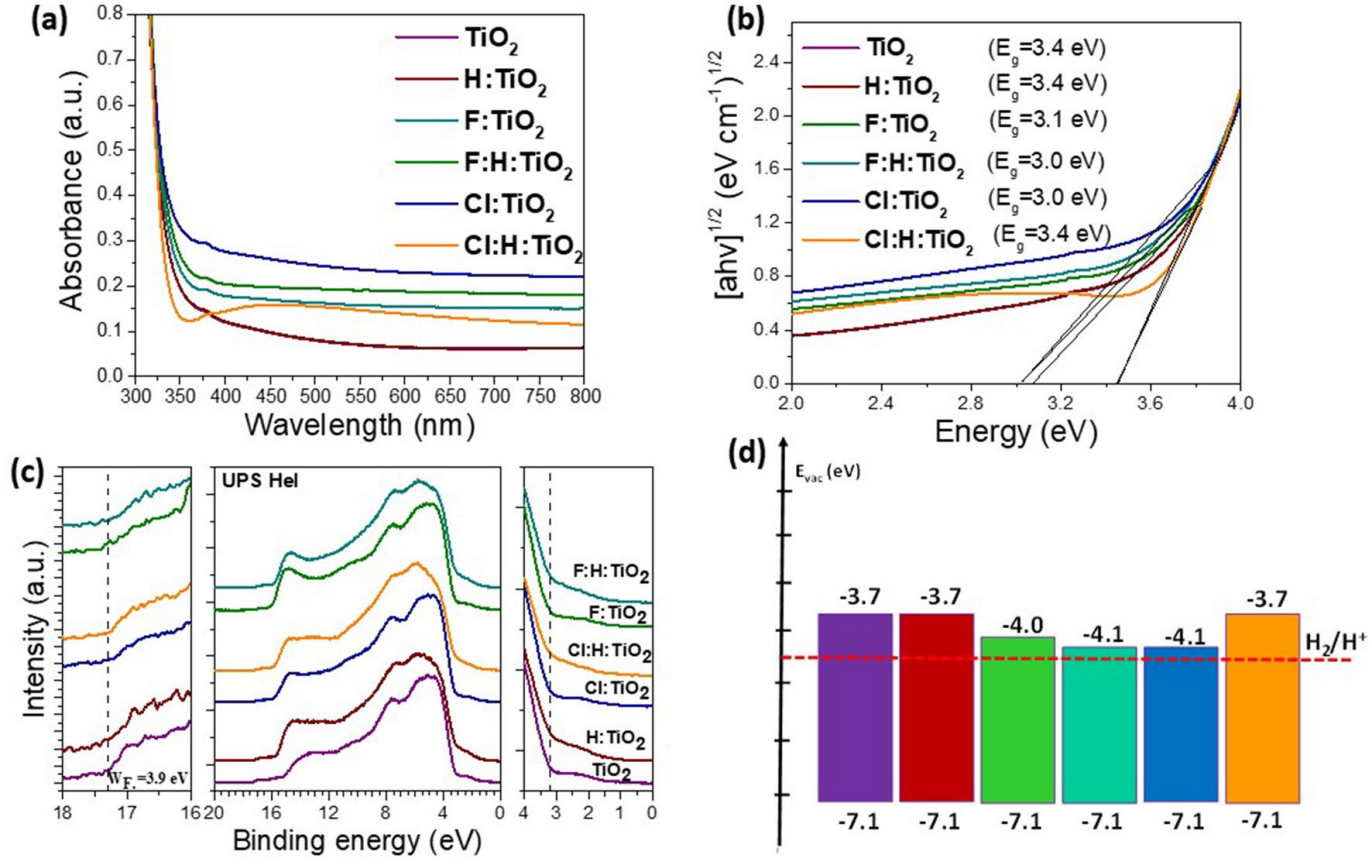
(**a**) UV–Vis absorption spectra and (**b**) the corresponding Tauc plots of all samples. (**c**) UP spectra (middle), the high BE cut-off (left) and the valence band maximum (VBM) (right) regions are presented for all the samples. (**d**) The electronic band structure of the TiO2 samples according to UP spectra and Tauc plots.

For the photocatalytic mechanism of the un-doped and doped samples to be fully understood, the optical properties are investigated. As it is presented in Fig. 5a, the UV–visible absorption spectra reveal that all these samples have an absorption range in the ultraviolet light region. Comparing to the un-doped TiO_2_ and the H:TiO_2_ samples, the F:TiO_2_ and the F:H:TiO_2_ have an increased UV light absorption. In the hydrogenation reaction of TiO_2_, O–Ti–H bonds were formed on the surface, which passivate the dangling bonds and affects the absorption spectra of the sample^70^. Hydrogen annealing actively enhances the visible light absorption of TiO_2_, which is consistent with other reports^71^. The same can also be observed in the Cl:TiO_2_ sample. In the case of Cl:H:TiO_2,_ however, it is observed that even though enhanced absorption within the visible is achieved relative to the pristine sample, hydrogen annealing reduces the visible absorption of the Cl doped sample. This is probably an indication that during hydrogen annealing some of the intercalated Cl atoms have left the oxide’s lattice. The band gap value of each sample was calculated from the corresponding Tauc plots, which are presented in Fig. 5b. It is clear that the bang gap for the hydrogenated samples is smaller than the pure TiO_2_. In the case of Cl:H:TiO_2,_ it is seen that the band gap is highly reduced, which is in agreement with the previous DFT calculations. From the above, it is demonstrated that hydrogenation treatment can improve the absorption capacity of TiO_2_. Moreover, although it is seen in the literature that H:F:TiO_2_ is an excellent photocatalyst, we present here evidence that Cl:H:TiO_2_ exhibits even better characteristics.

#### Electronic structures

In Fig. 5c, the ultra-violet photoelectron (UP) spectra (middle), the high BE cut-off (left) and the valence band maximum (VBM) (right) regions are presented for all the samples. Considering the work function (W_F_), as taken from the high BE cut-off, for the pristine TiO_2_ sample, is 3.9 ± 0.1 eV and no change is observed after the different treatments. The VBM for all the samples is measured from the intersection of the valence band cut-off with the background and is 3.2 ± 0.1 eV. The shoulder present around 2.5 eV is due to gap states induced by the O defects on the TiO_2_ surface and is greater for the H_2_ treated samples. For the samples after the H_2_ treatment, another feature around 6 eV can be seen and it is due to the presence of more adventitious carbon, as observed by the FTIR and XPS (not shown). Based on the UPS measurements and Tauc plots we constructed the band structure of the six TiO_2_ samples studied herein which is presented in Fig. 5d. The energy levels of a photocatalyst are of paramount importance as they provide indication about the efficiency of the photocatalytic process. To proceed the photocatalytic reaction, the irradiation of the photocatalyst with UV/visible light results in the excitation of electrons in the CB while holes are left in the VB. The electrons then reduce the H^+^ to hydrogen gas. In order for the latter process to be efficient the CB of the semiconductor should be close or even aligned with the hydrogen reduction potential (HRP, − 4.5 eV). It is therefore concluded that F,H co-doped and Cl doped TiO_2_ samples should be more effective in H^+^ reduction as their CB lies closer to HRP.

#### Photocatalytic hydrogen production

**Figure 6:**
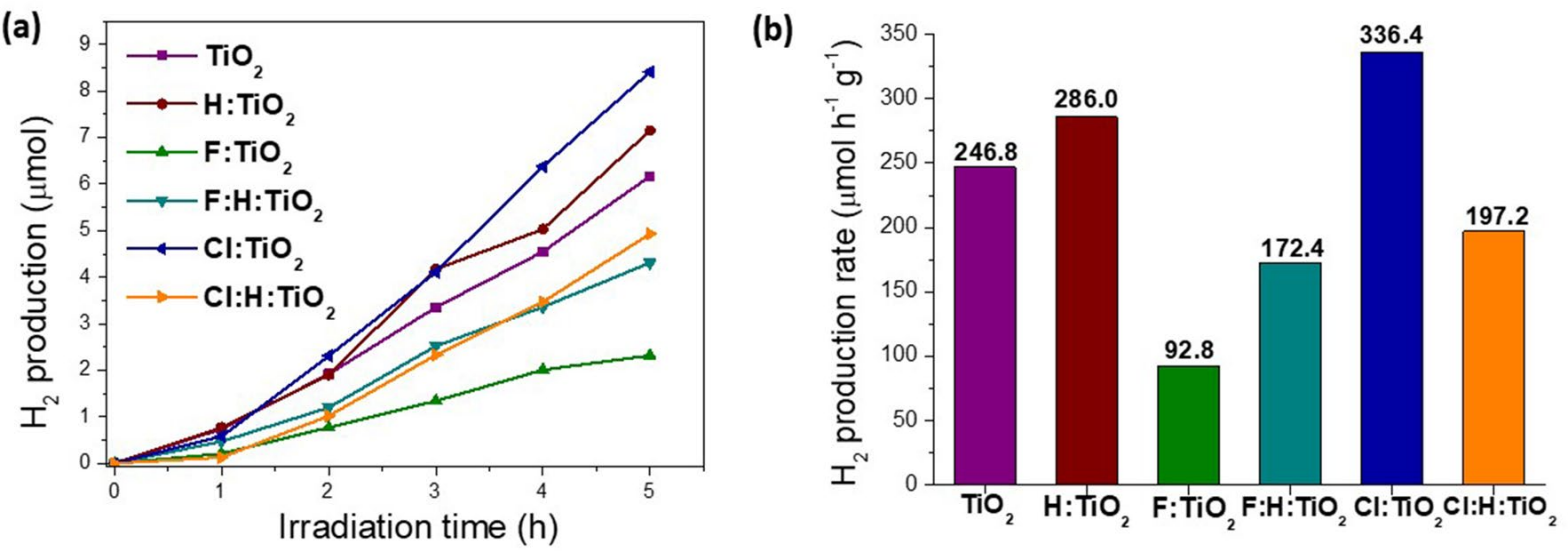
Photo-catalytic hydrogen production plots (**a**) and average rate (**b**) of different samples.

We next performed some preliminary studies on the photocatalytic performance of our TiO_2_ samples. The photocatalytic H_2_ production of all samples was examined under UV light (375 nm) irradiation. As presented in Fig. 6, all the TiO_2_ samples are capable to perform the photocatalytic proton reduction, since H_2_ evolution was observed in all cases. Cl:TIO_2_ presented the best H_2_ evolution activity (336.4 μmol h^−1^ g^−1^), while the lowest catalytic performance was observed for the F:TiO_2_ (92.8 μmol h^−1^ g^−1^). Moreover, with the exception of the chlorine doped TiO_2_, the hydrogen annealing improved the photocatalytic activity of the samples. Although these are preliminary studies, they however indicate that Cl doping, which has not been widely investigated, represent an affective route to enhance the photocatalytic activity of TiO_2_ and related materials.

## Conclusions

In this work, H, F and Cl doped and co-doped TiO_2_ samples have been successfully synthesized and studied both from a theoretical and experimental point of view. It is seen that halogen doping significantly reduces the band gap and provides a higher absorbance capacity to the oxide material. However, Cl doping results in maximum absorption within the visible which is also combined with a CBM located near the potential for hydrogen reduction. Our preliminary studies indicate higher hydrogen production in the Cl doped sample. The above results demonstrate that although there are many reports which propose the F:TiO_2_ as a better photocatalyst, the Cl:TiO_2_ one exhibits better characteristics, and it should be further examined for energy related applications.

## Methods

### Computational methodology

DFT calculations using the CASTEP program^72, 73^ were performed. For the exchange–correlation functional, the Perdew, Burke and Ernzerhof (PBE)^74^ generalized gradient approximation (GGA) functional was employed with ultrasoft pseudopotentials^75^. The cut-off Energy was converged at 480 eV and a 3 × 3 × 1 Monkhorst–Pack (MP)^76^k-points mesh for supercells of 108 atoms that were adopted for the bulk system. The structure was optimized with the Broyden–Fletcher–Goldfarb–Shanno (BFGS) method. To consider the effects of electron localization, the DFT + U method was employed for spin-polarized calculations with on-site Coulomb interactions of 8.2 eV for the 3d orbitals of Ti. Finally, for the DOS calculations, a 3 × 3 × 3 k-points mesh of was used, while for the PDOS a set of 7 × 7 × 7. For the surface structures, a supercell consisting of 96 atoms was optimized, with an energy cut off of 480 eV and a MP k-point mesh of 2 × 2 × 1. Finally, for the surface DOS, we chose a 7 × 7 × 7 k-point mesh.

### Experimental methods

The samples were prepared on glass which was cleaned by sonication in detergent solution, water, acetone and isopropyl alcohol. The solution 1 consisted of 13 μL concentrated aqueous HCl in 5 mL of dry isopropyl alcohol. The solution 2 consisted of titanium isopropoxide (711 mg, 2.5 mol) and 5 mL dry isopropyl alcohol was stirred for 30 min and then the solution 1 was slowly added to solution 2. The cleaned glass substrates were spin-coated at 2000 rpm and then placed on a hotplate of 150 °C. After 10 min the substrates were calcinated at 500 °C for 45 min. For the fluorine and chlorine dopants, 10 mg of NH_4_F and NH_4_Cl precursor was mixed with 1 mL water. The doped structures were formed by mixing 70 μL dopant with 700 μL TiO_2_. Hydrogen doped and co-doped structures were obtained after annealing with hydrogen gas at 500 °C for 30 min. The annealing was made in a furnace consisting of a quartz chamber with a graphite base for placing the samples. The base was heated by three tungsten halogen lamps of 1000 W each. For the temperature control, we used an automatic temperature control system, which was connected with a thermocouple on the graphite base, which controlled the power of the lamps. After loading the samples, the air pressure evacuated down to 3. 10^–2^ Torr. Afterward, nitrogen gas was allowed to flow in order to maintain a pressure of 0.1 Torr, and at this point, the temperature went up to the desired temperature of 500 °C. When the temperature was reached, the chamber was evacuated again to 3. 10^–2^ Torr, and the hydrogen gas was inserted in it at 1 Torr pressure. The duration of the hydrogen annealing of the samples was 30 min. In the end, the samples were cooled at 50 °C under 0.1 T of nitrogen gas. Fourier transform infrared (FTIR) transmittance spectra were obtained using a Bruker Tensor 27 FTIR spectrometer having a DTGS detector. X-ray diffraction (XRD) measurements were carried out with a Siemens D500 diffractometer with Cu-Ka radiation. A Perkin Elmer Lambda 40 UV–Vis spectrometer was used to record the absorption spectra of the samples. All the samples were introduced to the ultra-high vacuum chamber for XPS and UPS measurements as received. XPS measurements were performed using unnmonochromatized Al K*α* X-rays (1486.6 eV) and a hemispherical energy analyzer (Leybold EA-11) with a constant pass energy of 100 eV. The full width at half maximum for a reference Au 4f_7/2_ peak is 1.5 eV. The analyzed area was an approximately 2 × 5 mm^2^ rectangle positioned near the geometric center of each sample. XPS analysis was carried out at 0° take-off angle (normal to the sample surface). The C 1 s peak at 284.8 eV binding energy was used for surface electrostatic charging correction in all spectra. For the UPS measurements, He I line was used and a bias was applied between the sample and the analyzer in order to separate the secondary electrons.

### Photocatalytic H_2_ production studies

5 mg of each photocatalyst was dispersed in a mixture of 1 mL methanol and 4 mL ultrapure water and sonicated for 2 min. The samples were sealed with a septum and purged with nitrogen gas for 3 min in order to remove oxygen. Photocatalytic experiments were conducted with a 100 W UV led (375 nm) lamp, under continuously stirring. At certain time intervals, 100 μL was removed from the headspace of the flask and injected into a Shimadzu GC 2010 plus chromatograph with a TCD detector and a molecular sieve 5 Å column (30 m–0.53 mm) in order to measure the amount of H_2_ that was produced. The quantification of the produced H_2_ was done by using a calibration curve.
